# Supplementary material for: Transrenal Mycobacterium tuberculosis DNA in pulmonary tuberculosis patients during the first 14 days of treatment
Source: Microbiol Spectr. 2023 Oct 26;11(6):e02348-23. doi: 10.1128/spectrum.02348-23 (PMC10714760; doi:10.1128/spectrum.02348-23)
Supplement: Supplemental file 1 — Fig. S1 and S2 and Tables S1 to S4 [file spectrum.02348-23-s0001.doc]

**Online Supplementary Data**

Figure S1. Distribution of sputum culture with high bacterial load (TTP<7) by days of treatment in study arms. Mero - meropenem, Ert - ertapenem, Amx - amoxicillin, CA – clavulanate, Rif – rifampicin, IM – intramuscularly, IV – intravenously. Number in the brackets indicates number of study participants in each arm.


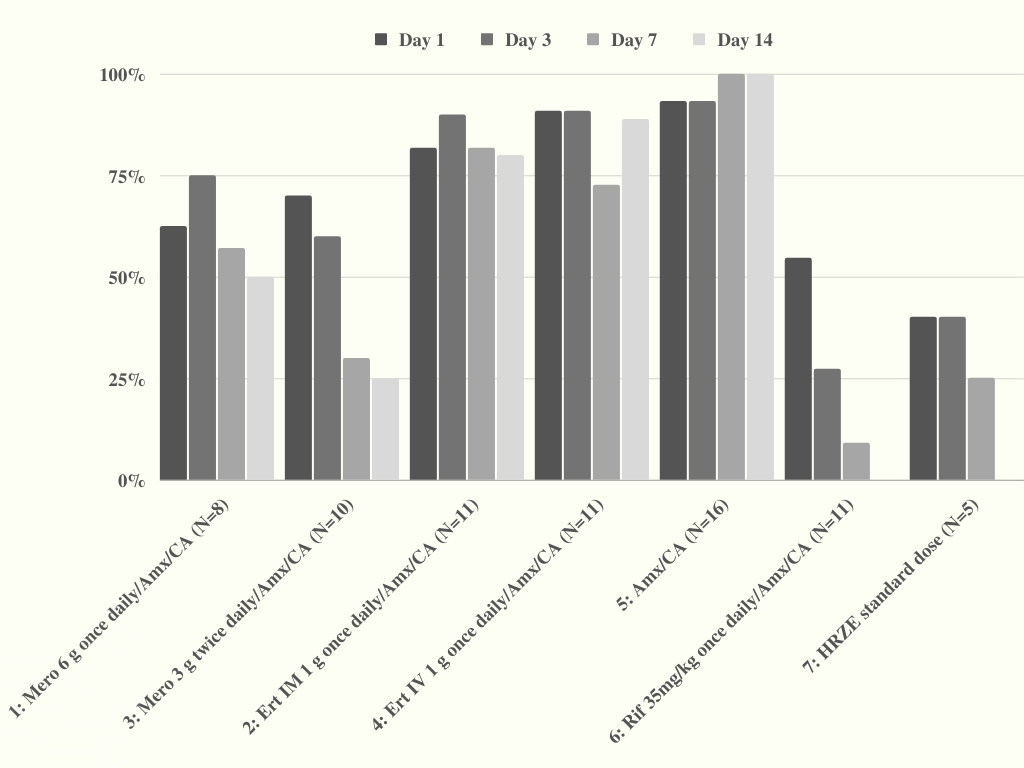


Figure S2. The proportion of positive PCR (Ct<36) for trDNA in urine taken in parallel with cultures with high (TTP<7d) and low (TTP≥7d) bacterial load. TTP – time to positivity.


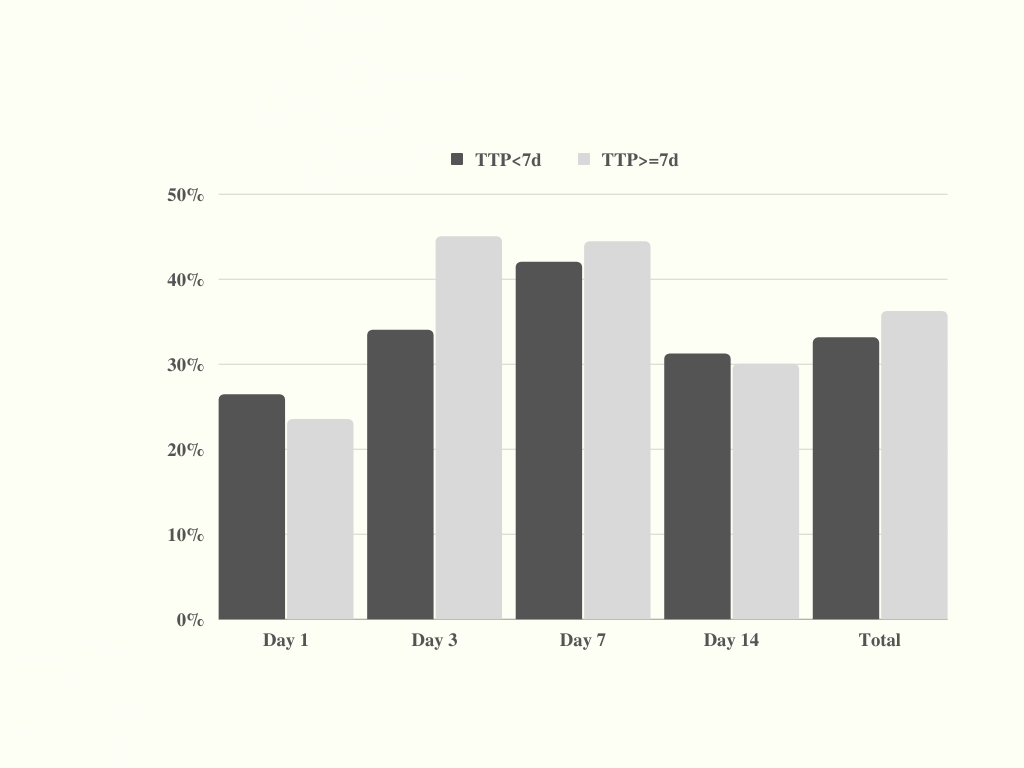

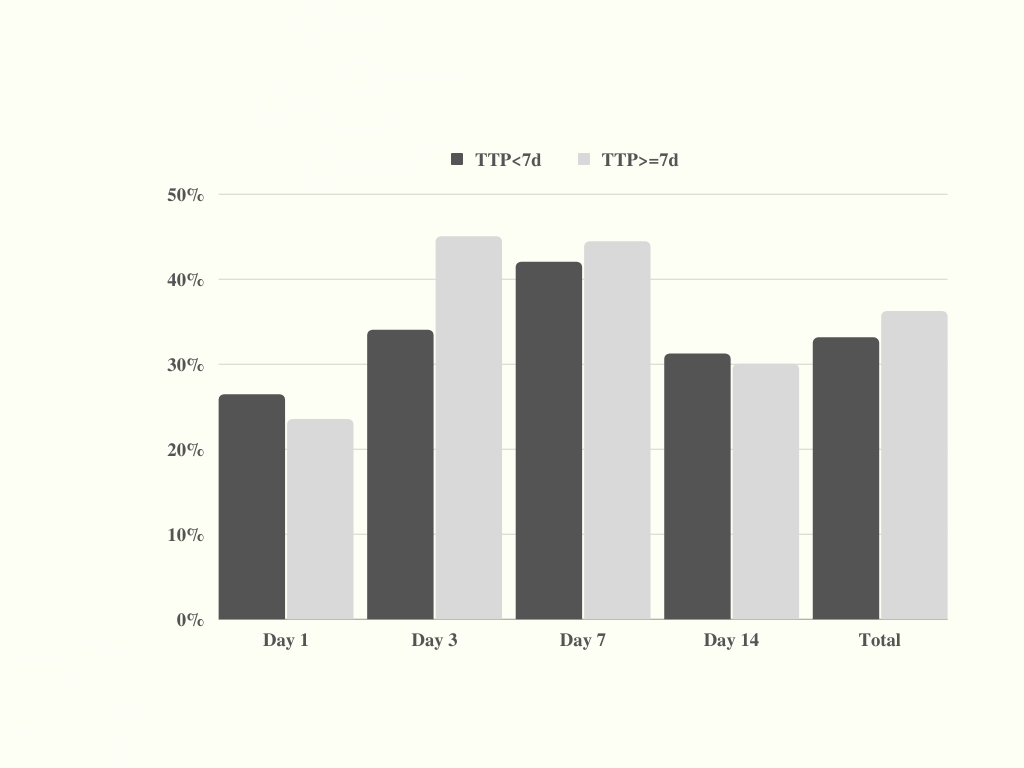


Table S1. The proportion of cultures with high (TTP<7d) and low (TTP≥7d) bacterial load over the course of treatment.

| Day of treatment | Number of cultures (n/N, %) | |
| --- | --- | --- |
|  | TTP<7d | TTP≥7d |
| Day 1 | 53/70 (75.7%) | 17/70 (24.3%) |
| Day 3 | 50/70 (71.4%) | 20/70 (28.6%) |
| Day 7 | 40/67 (59.7%) | 27/67 (40.3%) |
| Day 14 | 32/62 (51.6%) | 30/62 (48.4%) |

TTP – time to positivity.

Table S2. Distribution of sputum culture with high bacterial load (TTP<7) by day of treatment in study arms.

| Study arm | Number of cultures with TTP<7 (n/N, %) | | | |
| --- | --- | --- | --- | --- |
|  | Day 1 | Day 3 | Day 7 | Day 14 |
| Meropenem 6 g once daily/Amx/CA | 5/8 (62.5%) | 6/8 (75.0%) | 4/7 (57.1%) | 3/8 (50.0%) |
| Meropenem 3 g twice daily/Amx/CA | 7/10 (70.0%) | 6/10 (60.0%) | 3/10 (30.0%) | 2/8 (25.0%) |
| Ertapenem IM 1 g once daily/Amx/CA | 9/11 (81.8%) | 9/10 (90.0%) | 9/11 (81.8%) | 8/10 (80.0%) |
| Ertapenem IV 1 g once daily/Amx/CA | 10/11 (90.9%) | 10/11 (90.9%) | 8/11 (72.7%) | 8/9 (88.9%) |
| Amoxicillin/CA | 14/15 (93.3%) | 14/15 (93.3%) | 14/14 (100.0%) | 11/11 (100.0%) |
| Rifampicin 35mg/kg once daily/Amx/CA | 6/11 (54.5%) | 3/11 (27.3%) | 1/11 (9.1%) | 0/11 (0.0%) |
| HRZE standard dose | 2/5 (40.0%) | 2/5 (40.0%) | 1/4 (25.0%) | 0/5 (0.0%) |

Amx – amoxicillin, CA – clavulanate, HRZE – standard of care (isoniazid, rifampicin, pyrazinamide, ethambutol), IM – intramuscularly, IV – intravenously, TTP – time to positivity.

Table S3. The proportion of positive PCR results (Ct<36) for trDNA in urine taken in parallel with cultures with high (TTP<7d) and low (TTP≥7d) bacterial load.

| Day of treatment | Number of PCR positive samples (n, %) | | P-value |
| --- | --- | --- | --- |
|  | TTP<7 | TTP≥7d |  |
| Day 1 | 14/53 (26.4%) | 4/17 (23.5%) | 0.543 |
| Day 3 | 17/50 (34.0%) | 9/20 (45.0%) | 0.277 |
| Day 7 | 17/40 (42.5%) | 12/27 (44.4%) | 0.536 |
| Day 14 | 10/32 (31.2%) | 9/30 (30.0%) | 0.567 |
| Total | 58/175 (33.1%) | 34/94 (36.2%) | 0.356 |

TTP – time to positivity.

Table S4. trDNA PCR results and respective sputum culture results for study participants who had a positive (mean Ct<36) PCR result of trDNA assay on Day 1. Mero - meropenem, Ert - ertapenem, Amx - amoxicillin, CA – clavulanate, Rif – rifampicin, IM – intramuscularly, IV – intravenously, TTP – time to positivity, neg – negative, pos – positive, cont. – contaminated, n/a – not available. Positive PCR results (mean Ct<36) are in bold.

| Participant ID | Time point | Ct 1 | Ct 2 | Ct 3 | Ct mean | Culture 1 | TTP 1 | Culture 2 | TTP 2 | TTP mean | TTP<7 | Study arm |
| --- | --- | --- | --- | --- | --- | --- | --- | --- | --- | --- | --- | --- |
| T3-008 | Day 1 | 35.11 | 34.39 | 34.67 | **34.72** | pos | 04:07 | pos | 04:07 | 04:07 | yes | Ert IM 1 g once daily/Amx/CA |
|  | Day 3 | 34.40 | 34.02 | neg | **34.21** | pos | 04:04 | pos | 04:07 | 04:05 | yes | Ert IM 1 g once daily/Amx/CA |
|  | Day 7 | 33.40 | 33.00 | neg | **33.20** | pos | 04:00 | pos | 04:02 | 04:01 | yes | Ert IM 1 g once daily/Amx/CA |
|  | Day 14 | neg | neg | neg | neg | pos | 04:06 | pos | 04:03 | 04:04 | yes | Ert IM 1 g once daily/Amx/CA |
| T3-010 | Day 1 | neg | 33.43 | 32.57 | **33.00** | pos | 02:16 | pos | 02:11 | 02:13 | yes | Ert IM 1 g once daily/Amx/CA |
|  | Day 3 | neg | 34.13 | 34.69 | **34.41** | pos | 02:21 | pos | 03:01 | 02:41 | yes | Ert IM 1 g once daily/Amx/CA |
|  | Day 7 | 34.63 | 34.89 | 34.44 | **34.65** | pos | 03:19 | pos | 03:18 | 03:18 | yes | Ert IM 1 g once daily/Amx/CA |
|  | Day 14 | neg | 34.24 | 33.94 | **34.09** | pos | 03:02 | pos | 03:02 | 03:02 | yes | Ert IM 1 g once daily/Amx/CA |
| T3-013 | Day 1 | neg | 33.07 | neg | **33.07** | pos | 07:18 | pos | 07:17 | 07:17 | no | Mero 6 g once daily/Amx/CA |
|  | Day 3 | neg | neg | 33.54 | **33.54** | pos | 05:19 | pos | 05:19 | 05:19 | yes | Mero 6 g once daily/Amx/CA |
|  | Day 7 | 33.85 | 33.83 | 33.90 | **33.86** | pos | 05:12 | pos | 05:12 | 05:12 | yes | Mero 6 g once daily/Amx/CA |
|  | Day 14 | 34.72 | 34.25 | 34.63 | **34.53** | pos | 07:06 | pos | 07:06 | 07:06 | no | Mero 6 g once daily/Amx/CA |
| T3-035 | Day 1 | 35.14 | 35.18 | 35.01 | **35.11** | pos | 03:16 | pos | 03:15 | 03:15 | yes | Mero 6 g once daily/Amx/CA |
|  | Day 3 | 35.94 | 35.81 | 36.28 | 36.01 | pos | 04:10 | cont. | n/a | 04:10 | yes | Mero 6 g once daily/Amx/CA |
|  | Day 7 | 33.80 | 33.60 | 33.54 | **33.65** | cont. | n/a | pos | 07:07 | 07:07 | no | Mero 6 g once daily/Amx/CA |
|  | Day 14 | 33.89 | 34.10 | 34.01 | **34.00** | cont. | n/a | pos | 08:22 | 08:22 | no | Mero 6 g once daily/Amx/CA |
| T3-040 | Day 1 | 35.39 | 35.38 | 35.69 | **35.49** | n/a | n/a | n/a | n/a | n/a | n/a | Ert IM 1 g once daily/Amx/CA |
|  | Day 3 | 34.75 | 35.61 | 35.61 | **35.32** | pos | 03:19 | pos | 06:17 | 04:48 | yes | Ert IM 1 g once daily/Amx/CA |
|  | Day 7 | 35.49 | 36.44 | 36.58 | 36.17 | pos | 03:16 | pos | 03:18 | 03:17 | yes | Ert IM 1 g once daily/Amx/CA |
|  | Day 14 | 35.51 | 34.68 | 35.04 | **35.08** | pos | 04:19 | pos | 06:17 | 05:18 | yes | Ert IM 1 g once daily/Amx/CA |
| T3-041 | Day 1 | neg | 35.66 | 35.96 | **35.81** | pos | 11:08 | pos | 11:17 | 11:12 | no | HRZE standard dose |
|  | Day 3 | 35.65 | neg | 36.20 | **35.93** | pos | 11:09 | pos | 06:19 | 08:44 | no | HRZE standard dose |
|  | Day 7 | neg | 34.06 | 34.16 | **34.11** | pos | 14:23 | pos | 15:05 | 14:44 | no | HRZE standard dose |
|  | Day 14 | 36.45 | 36.12 | 35.98 | 36.18 | pos | 16:17 | pos | 17:23 | 16:50 | no | HRZE standard dose |
| T3-042 | Day 1 | 31.35 | 31.38 | 31.32 | **31.35** | pos | 04:15 | pos | 04:08 | 04:11 | yes | Mero 6 g once daily/Amx/CA |
|  | Day 3 | 35.67 | 35.68 | neg | **35.68** | pos | 05:14 | pos | 05:08 | 05:11 | yes | Mero 6 g once daily/Amx/CA |
|  | Day 7 | 34.70 | neg | neg | **34.70** | cont. | n/a | cont. | n/a | n/a | n/a | Mero 6 g once daily/Amx/CA |
|  | Day 14 | neg | neg | neg | neg | pos | 07:02 | pos | 07:09 | 07:05 | no | Mero 6 g once daily/Amx/CA |
| T3-044 | Day 1 | 34.83 | neg | neg | **34.83** | pos | 06:19 | pos | 06:17 | 06:18 | yes | Ert IM 1 g once daily/Amx/CA |
|  | Day 3 | neg | 36.48 | neg | 36.48 | pos | 06:20 | pos | 07:21 | 06:50 | yes | Ert IM 1 g once daily/Amx/CA |
|  | Day 7 | neg | neg | neg | neg | pos | 04:10 | pos | 03:13 | 03:41 | yes | Ert IM 1 g once daily/Amx/CA |
|  | Day 14 | neg | neg | neg | neg | pos | 04:09 | pos | 04:08 | 04:08 | yes | Ert IM 1 g once daily/Amx/CA |
| T3-054 | Day 1 | 35.12 | 35.22 | 35.32 | **35.22** | pos | 03:11 | pos | 03:13 | 03:12 | yes | Amx/CA |
|  | Day 3 | neg | neg | neg | neg | pos | 03:08 | pos | 04:21 | 03:44 | yes | Amx/CA |
|  | Day 7 | 36.21 | 35.78 | 35.90 | **35.96** | pos | 03:17 | pos | 03:18 | 03:17 | yes | Amx/CA |
|  | Day 14 | neg | neg | 35.59 | **35.59** | pos | 04:04 | pos | 03:20 | 03:42 | yes | Amx/CA |
| T3-056 | Day 1 | 34.71 | 34.93 | 34.52 | **34.72** | pos | 07:15 | pos | 09:05 | 08:10 | no | Ert IV 1 g once daily/Amx/CA |
|  | Day 3 | 35.90 | 36.24 | 36.12 | 36.09 | pos | 06:23 | pos | 08:20 | 07:21 | no | Ert IV 1 g once daily/Amx/CA |
|  | Day 7 | 33.79 | 34.14 | 33.78 | **33.90** | pos | 04:21 | pos | 05:04 | 04:42 | yes | Ert IV 1 g once daily/Amx/CA |
|  | Day 14 | 35.07 | 35.13 | 35.00 | **35.07** | n/a | n/a | n/a | n/a | n/a | n/a | Ert IV 1 g once daily/Amx/CA |
| T3-061 | Day 1 | neg | 35.58 | 35.49 | **35.54** | pos | 08:21 | pos | 09:14 | 08:47 | no | Mero 6 g once daily/Amx/CA |
|  | Day 3 | 35.04 | 34.52 | 35.13 | **34.90** | pos | 08:05 | pos | 08:11 | 08:08 | no | Mero 6 g once daily/Amx/CA |
|  | Day 7 | neg | neg | neg | neg | pos | 12:23 | pos | 13:17 | 12:50 | no | Mero 6 g once daily/Amx/CA |
|  | Day 14 | neg | neg | neg | neg | pos | 12:06 | pos | 12:06 | 12:06 | no | Mero 6 g once daily/Amx/CA |
| T3-077 | Day 1 | 33.31 | 32.95 | 33.04 | **33.10** | pos | 03:06 | pos | 03:06 | 03:06 | yes | Ert IV 1 g once daily/Amx/CA |
|  | Day 3 | 33.86 | 34.05 | 34.12 | **34.01** | pos | 03:13 | pos | 03:16 | 03:14 | yes | Ert IV 1 g once daily/Amx/CA |
|  | Day 7 | 33.54 | neg | neg | **33.54** | pos | 03:19 | pos | 03:20 | 03:19 | yes | Ert IV 1 g once daily/Amx/CA |
|  | Day 14 | neg | neg | 35.72 | **35.72** | pos | 05:04 | pos | 05:07 | 05:05 | yes | Ert IV 1 g once daily/Amx/CA |
| T3-078 | Day 1 | 33.95 | 33.84 | 33.90 | **33.90** | pos | 05:20 | pos | 05:19 | 05:19 | yes | Mero 3 g twice daily/Amx/CA |
|  | Day 3 | 34.42 | neg | neg | **34.42** | pos | 06:09 | pos | 06:16 | 06:12 | yes | Mero 3 g twice daily/Amx/CA |
|  | Day 7 | neg | neg | neg | neg | pos | 08:19 | pos | 08:15 | 08:17 | no | Mero 3 g twice daily/Amx/CA |
|  | Day 14 | neg | neg | neg | neg | pos | 11:06 | pos | 11:15 | 11:10 | no | Mero 3 g twice daily/Amx/CA |
| T3-079 | Day 1 | neg | neg | 35.92 | **35.92** | pos | 04:04 | pos | 04:04 | 04:04 | yes | Amx/CA |
|  | Day 3 | neg | neg | neg | neg | pos | 04:04 | pos | 04:02 | 04:03 | yes | Amx/CA |
|  | Day 7 | neg | 36.16 | neg | 36.16 | pos | 04:21 | pos | 04:23 | 04:22 | yes | Amx/CA |
|  | Day 14 | 36.26 | neg | 36.23 | 36.25 | cont. | n/a | cont. | n/a | n/a | n/a | Amx/CA |
| T3-086 | Day 1 | neg | neg | 34.62 | **34.62** | pos | 03:21 | pos | 03:19 | 03:20 | yes | Amx/CA |
|  | Day 3 | neg | neg | 36.27 | 36.27 | pos | 04:02 | pos | 04:05 | 04:03 | yes | Amx/CA |
|  | Day 7 | 35.31 | neg | 35.51 | **35.41** | pos | 03:12 | pos | 03:11 | 03:11 | yes | Amx/CA |
|  | Day 14 | neg | 35.67 | neg | **35.67** | pos | 03:13 | pos | 03:13 | 03:13 | yes | Amx/CA |
| T3-099 | Day 1 | neg | neg | 35.39 | **35.39** | pos | 05:05 | pos | 06:19 | 05:42 | yes | Rif 35mg/kg once daily/Amx/CA |
|  | Day 3 | neg | neg | neg | neg | pos | 05:23 | pos | 06:02 | 05:42 | yes | Rif 35mg/kg once daily/Amx/CA |
|  | Day 7 | neg | neg | neg | neg | pos | 07:01 | pos | 06:21 | 06:41 | yes | Rif 35mg/kg once daily/Amx/CA |
|  | Day 14 | neg | neg | 37.15 | 37.15 | pos | 09:14 | pos | 09:11 | 09:12 | no | Rif 35mg/kg once daily/Amx/CA |
| T3-109 | Day 1 | 35.02 | 34.95 | 34.65 | **34.87** | pos | 04:03 | pos | 04:05 | 04:04 | yes | Rif 35mg/kg once daily/Amx/CA |
|  | Day 3 | 33.04 | 33.43 | 33.54 | **33.34** | pos | 07:23 | pos | 07:21 | 07:22 | no | Rif 35mg/kg once daily/Amx/CA |
|  | Day 7 | 36.78 | 35.89 | 37.11 | 36.59 | pos | 12:20 | pos | 12:23 | 12:21 | no | Rif 35mg/kg once daily/Amx/CA |
|  | Day 14 | neg | neg | neg | neg | pos | 14:22 | pos | 15:23 | 14:52 | no | Rif 35mg/kg once daily/Amx/CA |
| T3-121 | Day 1 | neg | 34.34 | neg | **34.34** | pos | 04:04 | pos | 04:08 | 04:06 | yes | Mero 3 g twice daily/Amx/CA |
|  | Day 3 | 34.64 | neg | 34.47 | **34.56** | pos | 04:21 | pos | 04:16 | 04:18 | yes | Mero 3 g twice daily/Amx/CA |
|  | Day 7 | neg | neg | 35.34 | **35.34** | pos | 06:02 | pos | 06:17 | 06:09 | yes | Mero 3 g twice daily/Amx/CA |
|  | Day 14 | neg | neg | neg | neg | n/a | n/a | n/a | n/a | n/a | n/a | Mero 3 g twice daily/Amx/CA |
| T3-124 | Day 1 | neg | 34.67 | neg | **34.67** | cont. | n/a | cont. | n/a | n/a | n/a | Amx/CA |
|  | Day 3 | neg | neg | neg | neg | pos | 04:17 | pos | 04:17 | 04:17 | yes | Amx/CA |
|  | Day 7 | neg | neg | neg | neg | pos | 04:21 | pos | 04:19 | 04:20 | yes | Amx/CA |
|  | Day 14 | neg | neg | neg | neg | pos | 04:18 | pos | 04:18 | 04:18 | yes | Amx/CA |
| T3-133 | Day 1 | neg | neg | 32.15 | **32.15** | pos | 03:20 | pos | 03:18 | 03:19 | yes | Amx/CA |
|  | Day 3 | 32.64 | 32.57 | 32.46 | **32.56** | pos | 04:09 | pos | 04:09 | 04:09 | yes | Amx/CA |
|  | Day 7 | 32.68 | 32.86 | 32.68 | **32.74** | pos | 04:09 | pos | 04:03 | 04:06 | yes | Amx/CA |
|  | Day 14 | neg | neg | neg | neg | pos | 04:05 | pos | 04:04 | 04:04 | yes | Amx/CA |
